# Activities and Organophosphate Exposures: Need for the Numbers

**DOI:** 10.1289/ehp.112-a724b

**Published:** 2004-09

**Authors:** Robert I. Krieger, Xiaofei Zhang

**Affiliations:** Personal Chemical Exposure Program, Department of Entomology, University of California, Riverside, Riverside, California, E-mail: bob.krieger@ucr.edu

The article “Agricultural Task and Exposure to Organophosphate Pesticides among Farmworkers” (Coronado et al. 2004) seems to be founded on an erroneous premise and presents virtually no data to estimate levels of worker or child exposure. Useful data generated in conjunction with this research probably exists, but they were not published.

In the abstract, Coronado et al. (2004) state that

Little is known about pesticide exposure among farmworkers, and even less is known about the exposure associated with performing specific tasks.

The investigators open weakly by ignoring the substantial exposure (amount per person) data available related to work tasks of handlers [Pesticide Handlers Exposure Database; U.S. Environmental Protection Agency (EPA) 1995] and harvesters (U.S. EPA Transfer Coefficients) in the open literature and regulatory files of registrants and the U.S. EPA (U.S. EPA 1998).

We commend Coronado et al. (2004) for their use of a very large random sample of 213 farmworkers from 24 communities. The sensitive metabolite analyses of urine were reported as “percent detectable dimethyl metabolites” without reference to the total amounts measured in the various urine specimens. This is unacceptable for exposure assessment if their intent was, as they stated, to “examine the association between specific agricultural tasks and levels of exposure among adult workers and children living in the same household.” Failure to report urine metabolite levels deprives readers of the opportunity to transform percentages to dose, a measure of exposure. Dose (micrograms per person) defines the relationship of agricultural task to organophosphate (OP) exposure. Coronado et al. (2004) must have calculated the metabolite levels, but their failure to present those data seriously devalues the contribution and the cooperation of their subjects.

Coronado et al. (2004) reported the percentage of detectable dimethyl urinary metabolites in children (*n* = 211; 2–6 years of age). These data do not permit estimation of dose, and they prohibit full evaluation of the relationship of exposure from parents’ work tasks or other sources to the dimethyl metabolites from residential exposures, particularly diet (Krieger et al. 2003). It seems that the urine OP metabolite levels of children are more likely linked to dietary exposure (Zhang and Krieger 2004) than to environmental sources (Lowenhurz et al. 1997) proposed by Coronado et al. (2004). Meaningful discussion is again prohibited by the lack of metabolite urine levels presented.

The data presented by Coronado et al. (2004) are not adequate. We believe that the metabolite levels in urine should be published in *EHP* or otherwise made available to investigators.
